# *GmFT3a* fine-tunes flowering time and improves adaptation of soybean to higher latitudes

**DOI:** 10.3389/fpls.2022.929747

**Published:** 2022-07-25

**Authors:** Shan Yuan, Yining Wang, Junya Wang, Chunlei Zhang, Lixin Zhang, Bingjun Jiang, Tingting Wu, Li Chen, Xin Xu, Yupeng Cai, Shi Sun, Fulu Chen, Wenwen Song, Cunxiang Wu, Wensheng Hou, Lijie Yu, Tianfu Han

**Affiliations:** ^1^MARA Key Laboratory of Soybean Biology (Beijing), Institute of Crop Sciences, Chinese Academy of Agricultural Sciences, Beijing, China; ^2^College of Life Science and Technology, Harbin Normal University, Harbin, China

**Keywords:** soybean, *GmFT3a*, flowering time, photoperiod, adaptation

## Abstract

Onset of flowering of plants is precisely controlled by extensive environmental factors and internal molecular networks, in which *FLOWERING LOCUS T* (*FT*) is a key flowering integrator. In soybean, a typical short-day plant, 11 *FT* homologues are found in its genome, of which several homologues are functionally diversified in flowering pathways and the others including *GmFT3a* are yet unknown. In the current study, we characterized *GmFT3a*, which is located on the same chromosome as the flowering promoters *GmFT2a* and *GmFT5a*. Overexpression of *GmFT3a* significantly promoted flowering of Arabidopsis under the inductive long-day (LD) photoperiod. *GmFT3a* over-expressed soybean also flowered earlier than the control under LD, but they were not significantly different under inductive short-day (SD) conditions, indicating that *GmFT3a* acts as a flowering promoter in the non-inductive photoperiod in soybean. Compared with other *GmFT* homologues, *GmFT3a* exhibited a slighter effect in flowering promotion than *GmFT2a*, *GmFT5a* and *GmFT2b* under LD conditions. *GmFT3a* promoted flowering by regulating the expression of downstream flowering-related genes and also affected the expression of other *GmFTs*. According to the re-sequencing data, the regional distributions of two major haplotypes in 176 soybean varieties were analyzed. The varieties with *GmFT3a*-Hap2 haplotype matured relatively early, and relative higher expression of *GmFT3a* was detected in early maturing varieties, implying that Hap2 variation may contribute to the adaptation of soybean to higher latitude regions by increasing expression level of genes in metabolism and signaling pathways. The early flowering germplasm generated by overexpression of *GmFT3a* has potential to be planted at higher latitudes where non-inductive long day is dominant in the growing season, and *GmFT3a* can be used to fine-tune soybean flowering and maturity time and improve the geographical adaptation.

## Introduction

Precise timing of flowering is critical to the environmental adaptation and productivity of crops (Cockram et al., 2007; Nakamichi, 2015). Understanding the molecular mechanisms underlying reproductive transition is a prerequisite for improving regional adaptability in crop breeding (Blümel et al., 2015; Lin et al., 2021a). In Arabidopsis, photoperiod, vernalization, gibberellin (GA), age and autonomous pathways were confirmed to be involved in the modulation of flowering (Zeevaart, 2008; Blümel et al., 2015). Among the environmental factors, photoperiod acts as a major determinant signal for flowering in many plants (Thomas and Vince-Prue, 1997; He et al., 2020). In the photoperiodic pathway, *FLOWERING LOCUS T* (*FT*), a key photoperiod-regulated flowering integrator, encodes florigen which functions as a leaf-derived long-distance mobile signals and promotes floral transition (Corbesier et al., 2007; Wigge, 2011). Homologues of *FT* are highly conserved and promotive to flowering in diverse species (Tamaki et al., 2007; Pin et al., 2010; Park et al., 2014; Qin et al., 2017; Zhang et al., 2021).

Soybean is a typical short-day plant but is now widely grown in a wide range of latitudes from 53°N to 40°S with diverse daylength (Watanabe et al., 2012; Jia et al., 2014; Liu et al., 2022), resulting from rich varietal diversity in maturity. However, the cultivation area of a given variety is restricted to a narrow range of latitudes because of its sensitivity to photoperiods. In soybean breeding programs, the maturity or growth period which is mainly controlled by the photoperiod and temperature, is one of the most important traits for adaptation to a given environment (Hartwig, 1970; Wu et al., 2015; Song et al., 2019; Lin et al., 2021a).

Soybean maturity ecotypes were classified into a numerical and consecutive system “Maturity groups (MGs)” according to photothermal sensitivity and adaptation to specific environments (Hartwig, 1970; Jia et al., 2014; Mourtzinis and Conley, 2017; Song et al., 2019). The diversity of soybean varieties in MG and adaptation to different environments benefit from the variations and combinations of the genes in the photoperiod pathway (Jiang et al., 2014; Wang et al., 2015; Zhang et al., 2020; Liu et al., 2020a; Lin et al., 2021b). It is important to deeply understand the effect of allelic variations in maturity-related genes on the adaptation of soybean varieties to diverse geographic regions and farming systems. Soybean is a diploid species derived from an ancient tetraploid, and its genome has undergone whole-genome duplications during its long evolutionary history (Wang et al., 2015). Since then, many genes have multiple copies in the genome. For *FT* homologues, the integrators in the flowering pathway, there are at least 11 homologues in soybean (Kong et al., 2010; Liu et al., 2020b; Lee et al., 2021; Zhang et al., 2021). Among these genes, *GmFT2a* (*Glyma.16 g150700*) and *GmFT5a* (*Glyma.16 g044100*) play redundant but coordinated roles in flowering promotion by up-regulating the expression of floral determination genes (Kong et al., 2010; Sun et al., 2011; Na et al., 2013; Nan et al., 2014; Takeshima et al., 2019; Lin et al., 2021b). Moreover, *GmFT2b*, promotes flowering under long-days (LDs; Chen et al., 2020). *GmFT2b* overexpression plants exhibited early flowering under non-inductive LD conditions, while *ft2b* mutants flowered later than wildtype (WT) under LD. However, *GmFT1a* and *GmFT4* were identified as inhibiting genes for flowering. Overexpression of *GmFT1a* in soybean delayed flowering, confirming that it was a flowering repressor in soybean (Liu et al., 2018), and *GmFT4* was proven to be a repressor in Arabidopsis (Zhai et al., 2014). *GmFT3b* acts redundantly in flowering time regulation and may be compensated by other *FT* homologs in soybean (Su et al., 2022). The existence of multiple soybean *GmFT* homologues might enhance the adjustability of photoperiodic regulation of flowering (Jiang et al., 2019; Lin et al., 2021b). For instance, the expression of FT-like proteins is not restricted to short-day (SD) conditions (Kobayashi and Weigel, 2007), highlighting the coincident expression of FT-like genes encoding both floral activators and floral inhibitors in the day-neutral species of *Solanum lycopersicum* (Cao et al., 2016a) and *Nicotiana tabacum* (Beinecke et al., 2018). However, some important *GmFT* homologues have yet to be elucidated, which hinders a better understanding of the mechanism of functional allocations of the *GmFT* family in the photoperiodic flowering pathway. In this study, we focused on the *GmFT3a* (*Glyma.16 g044200*) which is highly homologous to *GmFT3b*, and investigated its function in the flowering control of soybean. These results will deepen the understanding of the functional diversification of *GmFT* members and provide a new target gene for improving the adaptation of soybean to diverse regions.

## Materials and methods

### Plant materials and growth conditions

For gene cloning of *GmFT3a*, Zigongdongdou (ZGDD), a late-maturing (MG IX) and photoperiod-sensitive soybean [*Glycine max* (L.) Merr.] variety which originated from Sichuan province in the southwest China (Wu et al., 2006) was selected as the plant material. Heihe 27 (HH27), an early maturing (MG 00) and photoperiodic-insensitive and early maturing variety (MG 00) from Heilongjiang province in northeast China, was selected to compare the gene expression pattern with that of ZGDD. For genetic transformation, a medium-maturing (MG III) variety Jack was used as the receptor. Moreover, 12 MG standard soybean varieties were chosen for gene expression analysis of *GmFT3a*. All of soybean seeds were germinated in pots with soil and vermiculite (1:1) and the plants were grown in growth chambers with constant temperature (28°C) and controlled photoperiods, i.e., LD (16 h light/8 h dark) and SD (12 h light/12 h dark), respectively. A diverse panel of 176 soybean cultivars with consecutive MG groups covering early MG 000 to MG IX, were re-sequenced to investigate the natural variation of *GmFT3a*. The germplasms were collected from China and the United States (Supplementary Table S2; Zhang et al., 2019; Liu et al., 2020c).

### *GmFT3a* gene cloning

Total RNA was extracted for gene cloning and expression analysis from the first trifoliolate leaf of ZGDD in LD (16 h light/8 h dark) using TransZol UP kit (TransGen Biotech, Beijing, China). The first strand of cDNA was obtained using the TransGene reverse transcription kit (TransGen Biotech, Beijing, China). The corresponding primer sequences are listed in Supplementary Table S1.

### Gene expression analysis

ZGDD, HH27 and 12 maturity group (MG) standard varieties (Supplementary Table S2) were treated with LD and SD, respectively. After photoperiodic treatments (LD and SD) for 13 days, various tissues (shoot apex, unifoliolate, trifoliolate, stem, hypocotyl, root and flower) were taken to evaluate the expression level of *GmFT3a*. The real-time quantitative PCR (qRT-PCR) primers were designed by NCBI Primer Blast online with the amplification products ranging from 100 to 300 bp. Three-step PCR method was set with *GmActin* as the internal reference on the ABI Prism^®^ 7900HT real-time PCR instrument, and that relative expression of target genes was calculated according to the 2^-ΔΔCT^ algorithm (Pin and Nilsson, 2012).

### Subcellular localization of GmFT3a protein

The subcellular localization of GmFT3a protein was conducted by constructing p16318-*GmFT3a*-GFP fusion expression vector and carrying out plasmolysis isolation, transformation and culture of Arabidopsis protoplasts. The fusion *GmFT3a*-p16318- GFP subcellular localization vector of the experimental group and the empty p16318 vector of the control group were prepared and transformed into Arabidopsis protoplasts, and the expression position of GFP was observed in confocal laser scanning microscope (Olympus FV31-SD, Olympus Corporation, Japan).

### Expression vector construction and plant transformation

A fragment with *Xba*I and *Kpn*I digestion sites was ligated into pCAMBIA1300 and pTF101.1 vector to construct overexpression vectors, and the constructed vectors were transformed into Arabidopsis Columbia-0 by *Agrobacterium tumefaciens* mediation and inflorescence dipping method (Clough and Bent, 1998). The soybean variety Jack was used for transformation according to a previously published protocol (Chen et al., 2018). The transformed Arabidopsis plants were screened by hygromycin (Hyg), and the soybean plants were screened by applying 160 mg/l glufosinate solution on young leaves, and PCR identification.

### Transcriptome sequencing and data analysis

Leaf was sampled at 13 days after emergence (DAE) under SD conditions and 30 DAE under LD conditions. Three biological replicates were analyzed. The total amount of 1.5 μg RNA per sample was used as input material for the RNA sample preparations. Sequencing libraries were generated using NEBNext Ultra^™^ RNA Library Prep Kit for Illumina (NEB, United States) following manufacturer’s manual. In order to select cDNA fragments of 150 ~ 200 bp in length preferentially, the library fragments were purified with AMPure XP system (Beckman Coulter, Beverly, United States). The clustering of the index-coded samples was performed on a cBot Cluster Generation System using TruSeq PE Cluster Kit v3-cBot-HS (Illumina) according to the manufacturer’s instructions. The raw data have been uploaded to NCBI Sequence Read Archive with the accession number of PRJNA832118.

All the downstream analyses were based on clean data with high quality. Gene function was annotated based on the following databases: Nr (NCBI non-redundant protein sequences); Nt (NCBI non-redundant nucleotide sequences); Pfam (Protein family); KOG/COG (Clusters of Orthologous Groups of proteins); Swiss-Prot (A manually annotated and reviewed protein sequence database); KO (KEGG Ortholog database); GO (Gene Ontology). Differential expression analysis of two conditions/groups was performed using the DESeq R package (1.10.1; Li and Dewey, 2011). DESeq provided statistical routines for determining differential expression in digital gene expression data using a model based on the negative binomial distribution. Genes with an adjusted *p* value <0.05 found by DESeq were assigned as differentially expressed. Gene Ontology (GO) enrichment analysis of the differentially expressed genes (DEGs) was implemented by the GOseq R packages-based Wallenius non-central hyper-geometric distribution (Young et al., 2010), which can adjust for gene length bias in DEGs. We used KOBAS (Mao et al., 2005) software to test the statistical enrichment of differential expression genes in KEGG pathways.

### Phenotyping and statistical analysis

Transgenic plants were grown in culture rooms under SD and LD conditions, respectively, and the flowering time was determined as the number of days from VE (emergence) to R1 stage (beginning bloom: the first flower appears at any node in the main stem; Fehr and Caviness, 1977). The statistical analysis was carried out by Microsoft Excel, and Student’s t-test was used to assess the significance of difference between the transgenic lines.

### Natural variations and haplotype identification

In order to evaluate the effects of *GmFT3a* natural variations on the adaptability of soybean varieties, the *GmFT3a* alleles of 176 soybeans germplasm were detected. The genomic data were obtained in our previous investigation (Zhang et al., 2019; Liu et al., 2020c) and the MG classification were carried out according to the standard methodology described by Song et al. (2019).

## Results

### Bioinformatic characteristics and subcellular localization of GmFT3a

**Figure 1:**
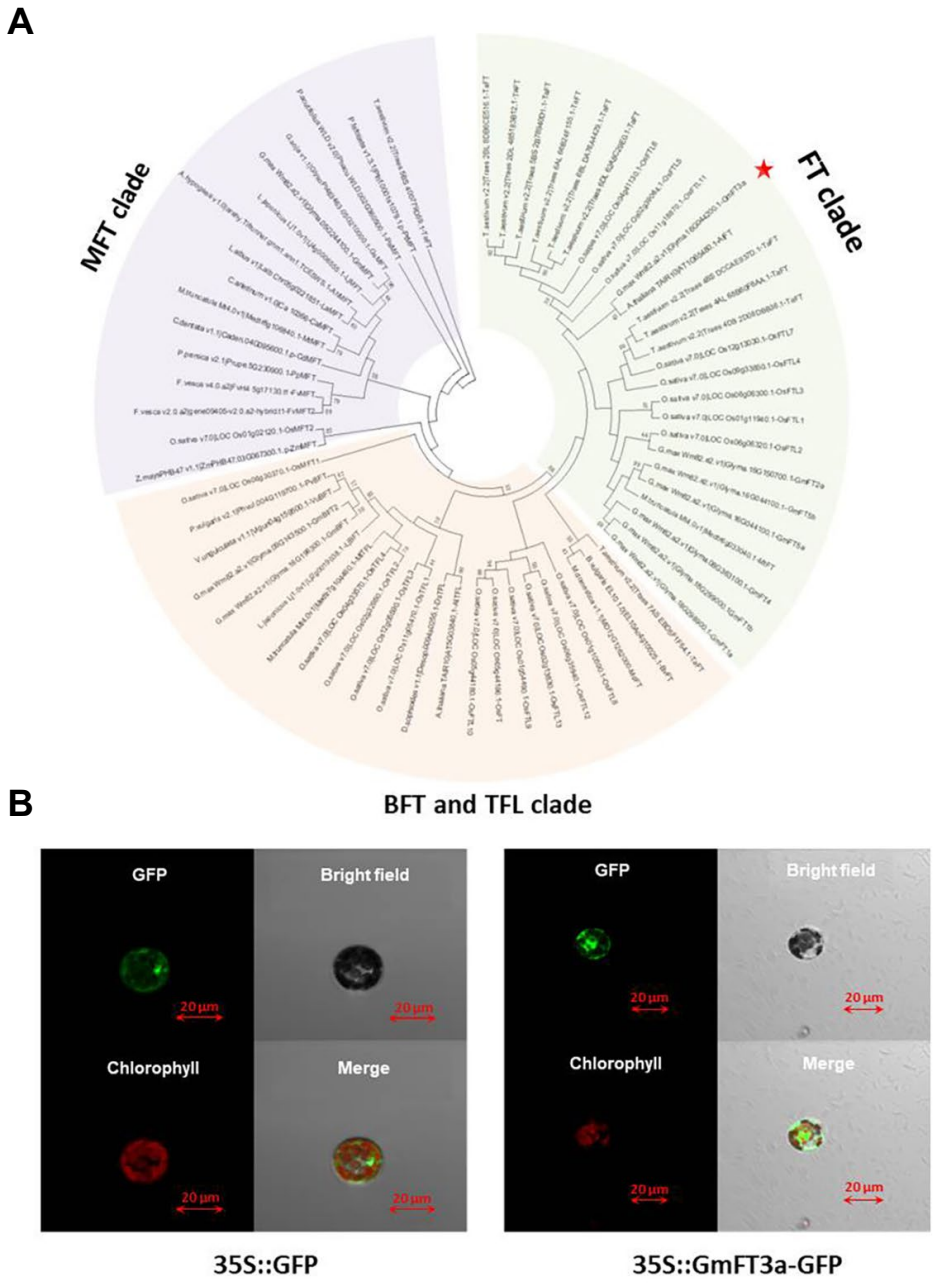
Phylogenetic tree based on protein sequences between *GmFT3a* and other FT/TFL1 family members from flowering plants **(A)** and subcellular localization of GmFT3a-GFP fusion protein in Arabidopsis protoplasts. The left four panels are 35S::GmFT3a-GFP constructs, and the right four panels are controls (35S::GFP) **(B)**.

The length of the *GmFT3a* CDS region is 528 bp in ZGDD, encoding 175 amino acids, which is a hydrophilic non-secretory protein. The region ranging from amino acid 27 to 164 is the PEBP domain (Supplementary Figures S1, S2). The phylogenetic tree divided these proteins into three clear groups based on their protein sequence homology, including (1) FT, (2) MFT (MOTHER of FT and TFL 1) and (3) BFT (BROTHER of FT and TFL 1) and TFL 1 (TERMINAL FLOWER 1) clades. GmFT3a was closely clustered with AtFT (AT1G65480.1; Figure 1A). The tertiary structure of its protein is similar to that of the AtFT protein and GmFT2a protein (Supplementary Figure S3). p16318-GFP in the control group did not show subcellular expression specificity, while fluorescence signals of GmFT3a-GFP were detected in cytoplasm and nucleus (Figure 1B). This result is similar to the subcellular localization of other functional phosphatidylethanolamine-binding proteins in soybean (Wang et al., 2015).

### *GmFT3a* expression was regulated by photoperiods and correlated with maturity groups of varieties

*GmFT3a* expression was evaluated in different tissues of the photoperiod-sensitive variety ZGDD. Under both SD and LD conditions, relatively higher expression was detected in trifoliolate, followed by the shoot apex and unifoliolate, and extremely lower expression was detected in stem, hypocotyl, root and flower tissues in ZGDD (Supplementary Figure S4a). The expression of *GmFT3a* was not significantly different among trifoliolate, shoot apex, and unifoliolate under SD, while the expression of trifoliolate was significantly higher than that of shoot apex and unifoliolate under LD and exceeded the average expression under SD (Supplementary Figure S4a).

We also analyzed the diurnal expression patterns of *GmFT3a* in trifoliolates of both HH27 and ZGDD since 13 DAE. In early variety HH27, and the consecutive 48 h sampling by every 4 h showed two major expression peaks at 4 h and 32 h in SD condition, but relative lower expression under LD, and only one peak on 0/48 h (Supplementary Figure S4b). In late variety ZGDD, the consecutive 48 h sampling every 4 h showed two peaks at 40 h and 4 h under LD, and no significant diurnal rhythm was displayed under SD (Supplementary Figure S4b). Under LD condition, the *GmFT3a* expression pattern of ZGDD was similar as under SD, but the highest expression was detected in the unifoliolate value in HH27, then followed by the trifoliolate and shoot apex (Supplementary Figure S4a).

Among the varieties belonging to different maturity groups, it was found that the expression of *GmFT3a* in the trifoliolates of early maturing groups of MG 000, MG 0 and MG VII was higher than that of the late-maturity groups under LD, while MG 000 and MG 00 maintained higher *GmFT3a* expression than the others under SD conditions (Supplementary Figure S4c).

### Overexpression of *GmFT3a* promoted flowering and regulated down-stream genes in both Arabidopsis and soybean

**Figure 2:**
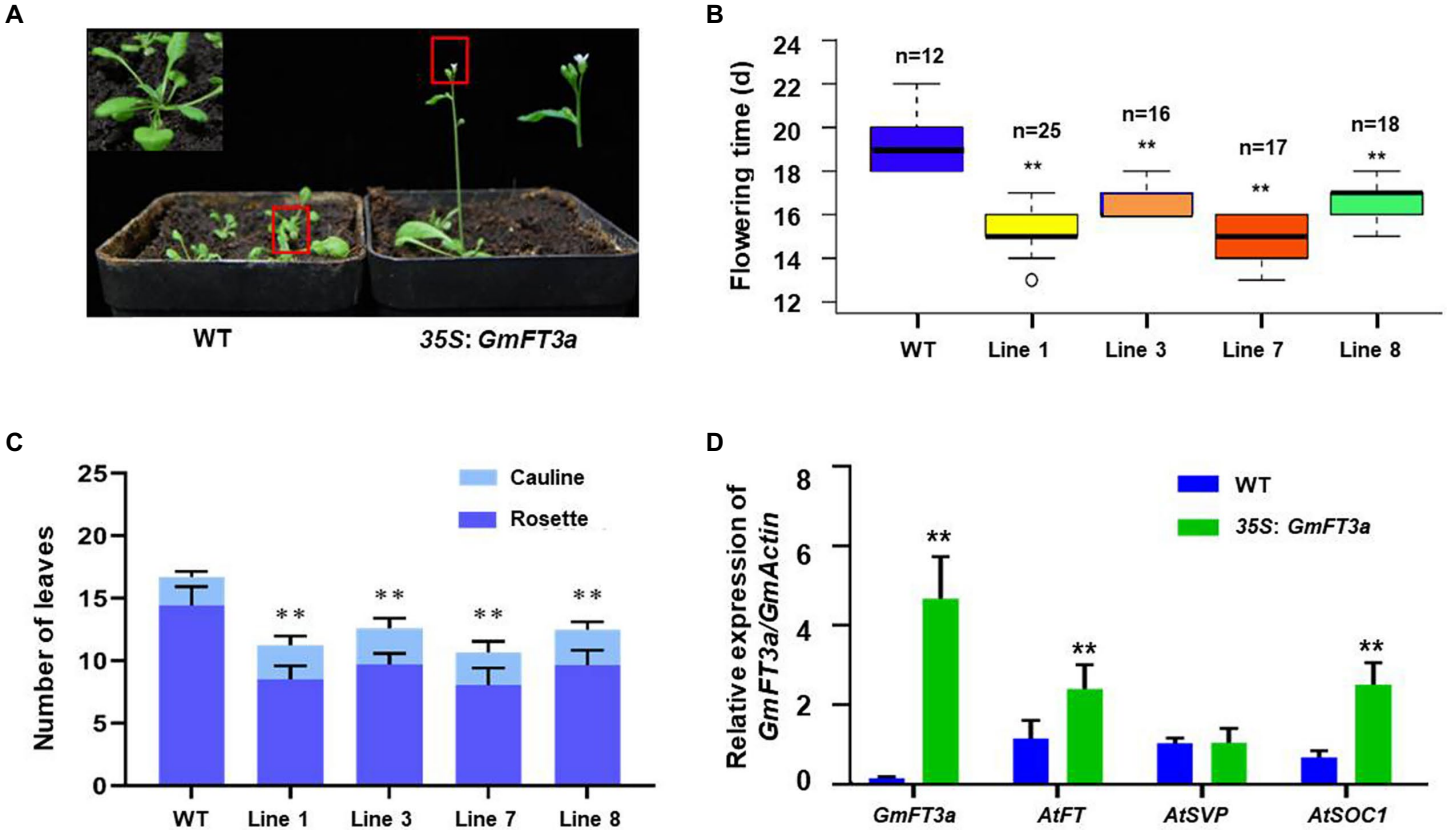
The phenotype **(A)**, flowering time **(B)**, the number of rosette and cauline rosette leaves at flowering **(C)** and the expression of flowering regulatory genes **(D)** of the transgenic *GmFT3a* lines under LD condition in Arabidopsis.

A total of four independent transgenic lines overexpressing *GmFT3a* were obtained in Arabidopsis, and an the obvious early flowering phenotype was observed in the T_5_ homozygous generation under LD treatment (Figure 2A). The average flowering time of the four lines was 3.9 d earlier than that of WT Arabidopsis (*p* < 0.01, Figure 2B). The average total (rosette and cauline) leaves of WT Arabidopsis were 16.7 ± 0.6, and those of the *GmFT3a* Arabidopsis overexpressing lines were 11.2 ± 1.0, 12.6 ± 1.3, 10.7 ± 1.4 and 12.5 ± 0.7, respectively. The average total leaf number of four lines was reduced by 29.6% compared with that of the WT (*p* < 0.01). Overexpression of *GmFT3a* resulted in a very significant early flowering phenotype (*p* < 0.01;s Figure 2C) in Arabidopsis. The qRT-PCR results showed that the ectopic *GmFT3a* caused significantly high expression of *AtFT*, *AtSOC1* and *AtCO* (*p* < 0.01, Figure 2D), and the up-regulation of the *AtFT* and *AtCO* in the photoperiod pathway might be the underlying reason for early flowering of transgenic *GmFT3a* Arabidopsis.

**Figure 3:**
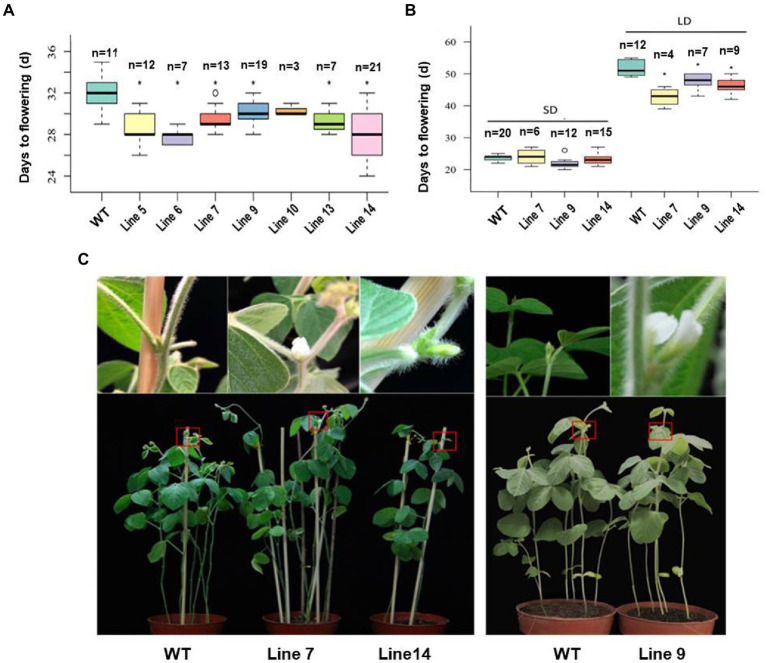
The flowering time of the transgenic *GmFT3a* soybean in natural **(A)** and long-day (LD) and short-day (SD) **(B)** conditions and the flowering phenotypes of transgenic *GmFT3a* T_2_ generation in LD condition **(C)**. The seeds sown in Beijing, China (N39°58′, E116°20′) on June 20, 2018. The data represent the mean ± standard deviation, and statistical significance was determined using Student’s *t*-tests (^*^p < 0.05, ^**^p < 0.01). DAE: days after emergence.

The fusion expression vector of *GmFT3a* was constructed and the variety Jack was used for *Agrobacterium*-mediated transformation. According to the flowering time of T_1_ generation and the number of seeds harvested, we selected three (Line 7, Line 9 and Line 14) from the *GmFT3a* overexpression lines (Figure 3A). In the T_2_ generation, the average flowering time of the Jack variety was 23.7 ± 1.5 DAE under SD, and that of the three transgenic *GmFT3a* lines were 21.7 ± 3.3, 21.5 ± 1.0 and 22.6 ± 3.7 DAE, respectively (Figure 3B). Under LD, the average flowering time of WT was 51.7 ± 4.1 DAE, while the three transgenic *GmFT3a* lines were 8.9 d, 4.0 d and 5.6 d earlier than that the WT, respectively (Figures 3B,C).

**Figure 4:**
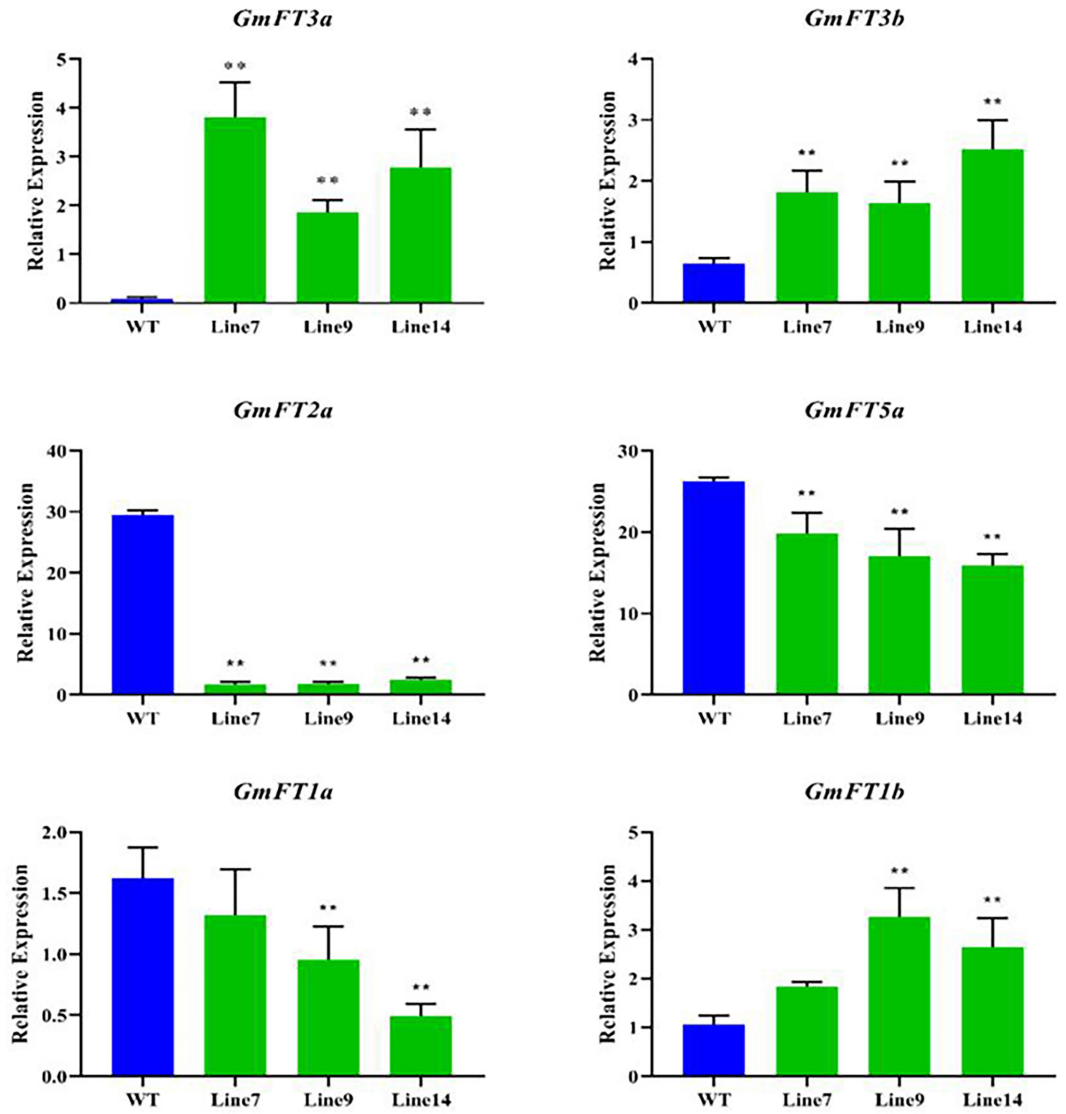
Expression analysis of *GmFT3a* in transgenic plants in LD condition.

The expression of *GmFT* homologues was measured in the transgenic *GmFT3a* soybean under LD condition, and their influence on other *GmFT* genes was analyzed. The results showed that the expression level of transgenic *GmFT3a* plants was significantly higher than that of WT (*p* < 0.01), and the expression level of *GmFT3b*, which has the highest homology with *GmFT3a*, was also significantly higher (*p* < 0.01), while the expression of *GmFT2a* and *GmFT5a* decreased significantly in the transgenic *GmFT3a* soybean (*p* < 0.01). The declining degree of *GmFT2a* expression was higher than that of *GmFT5a*. The expression of *GmFT1a* significantly decreased, while *GmFT1b* increased significantly in transgenic Line 9 and Line 14 (*p* < 0.01; Figure 4).

### More genes in metabolism and signaling pathways were enriched under LD than under SD among DEGs in transgenic *GmFT3a* soybean

**Figure 5:**
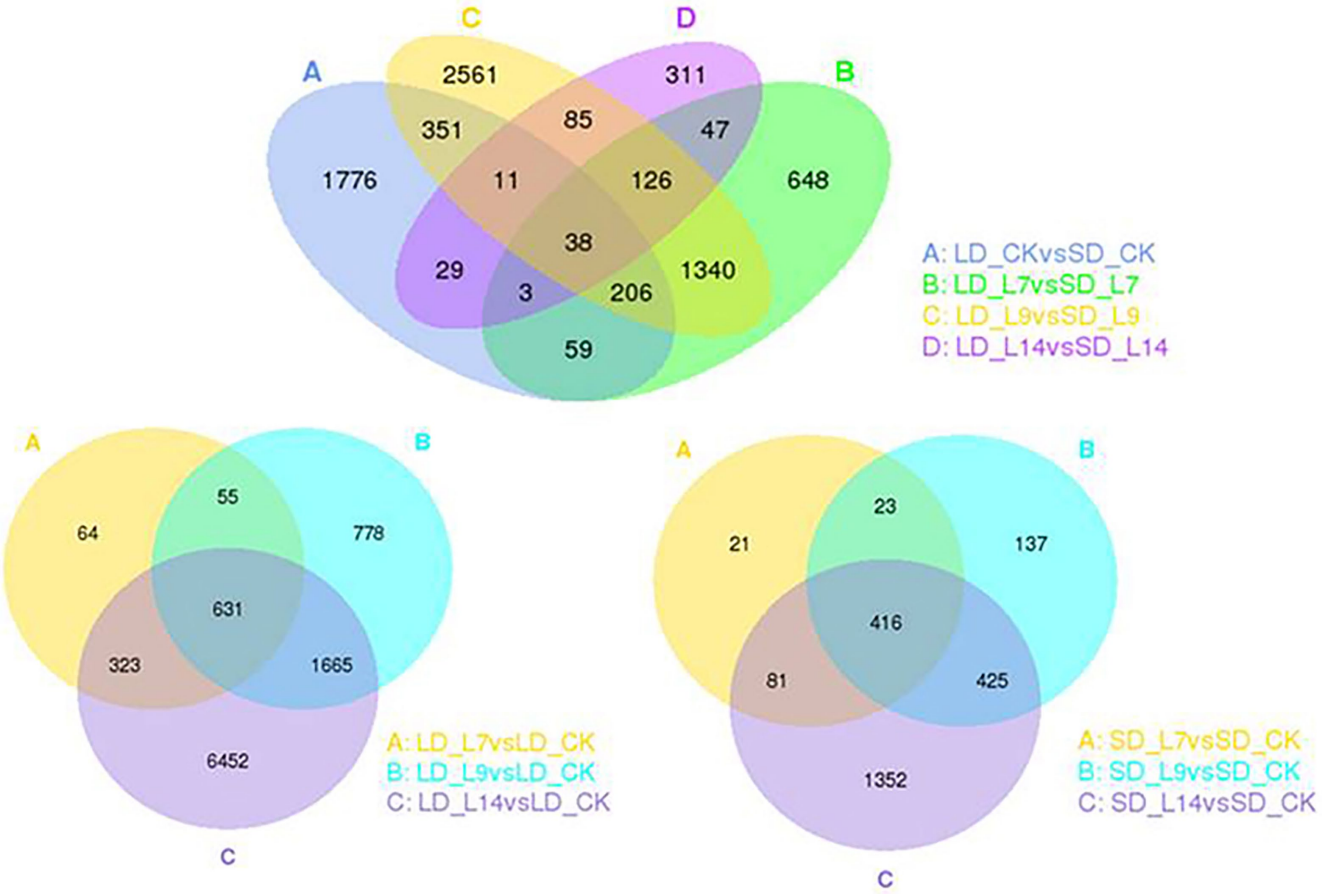
The Venn diagram of DEGs between transgenic *GmFT3a* soybean and control in LD and SD, respectively.

According to the transcriptomic profiles, a total of 3,129 DEGs with 1906 up-regulated and 1,223 down-regulated unigenes were observed under LD, and 1,001 DEGs including 489 up- and 512 down-regulated unigenes were expressed under SD (Table 1; Figure 5). Among these DEGs, there were 226 (153 up- and 73 down-regulated) unigenes under both LD and SD conditions (Table 1; Figure 5). Among the up-regulated unigenes under LD, 558 up-regulated genes were identified in the transgenic *GmFT3a* soybean (*p* < 0.05). These genes included F-box protein (a gene for the controlled degradation of cellular protein); disease resistance protein (a resistance protein guard the plant against pathogens); abscisic stress-ripening protein 3 (an abscisic acid-, stress-, and ripening-induced protein); heat shock protein 83 (a gene for promoting maturation), structural maintenance and proper regulation of specific target proteins involved for instance in cell cycle control and signal transduction.

**Table 1:** DEGs between transgenic *GmFT3a* soybean and control in LD and SD conditions.

Group	Up	Down	Total
LD	1906	1,223	3,129
SD	489	512	1,001
Specifically differed in LD	1753	1,150	2,903
Specifically differed in SD	354	421	775
Common	153	73	226

**Figure 6:**
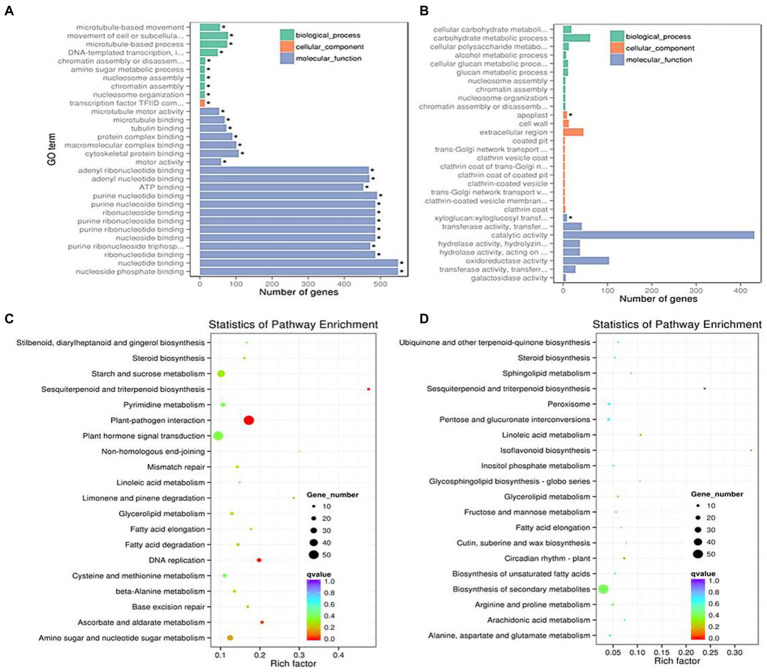
The most enriched GO terms in LD **(A)** and SD **(B)** and KEGG pathway terms in LD **(C)** and SD **(D)** conditions.

For further identifying the gene function, 49 and 143 GO terms were annotated in the up- and down-regulated DEGs, respectively under LD (Table 2). Under SD condition, only 10 GO terms significantly enriched in the down-regulated DEGs (corrected *p* < 0.05). Among the 49 up-regulated GO items, the “glucosamine-containing compound metabolic process,” “fatty acid biosynthetic process,” “signal transduction,” and “cellular protein modification process” items were significantly overrepresented in biological process; Other terms, such as “proteinaceous extracellular matrix,” “extracellular matrix,” “extracellular region part” were in cellular component, and “oxidoreductase activity, acting on NAD(P)H, oxygen as acceptor,” “kinase activity” and “ATP binding” were in molecular function. While under SD condition, no GO items were significantly enriched in the up-regulated DEGs and only 10 GO items were enriched in the down-regulated DEGs. The top 20 obviously enriched KEGG pathways are shown in Figure 6. By comparing transgenic *GmFT3a* group with control, “Plant hormone signal transduction” and “Amino sugar and nucleotide sugar metabolism” pathway enriched the most in the DEGs under LD condition; while only the “Biosynthesis of secondary metabolites” and “Sesquiterpenoid and triterpenoid biosynthesis” pathway were enriched under the SD condition (Figure 6).

**Table 2:** GO items between transgenic *GmFT3a* soybean and control in LD and SD conditions.

Group	Type	Biological process	Cellular component	Molecular function	Total
LD	Up	20	3	26	49
	Down	75	41	27	143
	Total	18	12	29	59
SD	Up	0	0	0	0
	Down	5	2	3	10
	Total	0	1	1	2

### Natural variations of *GmFT3a* existed in soybean varieties with diverse maturity groups and origins

**Figure 7:**
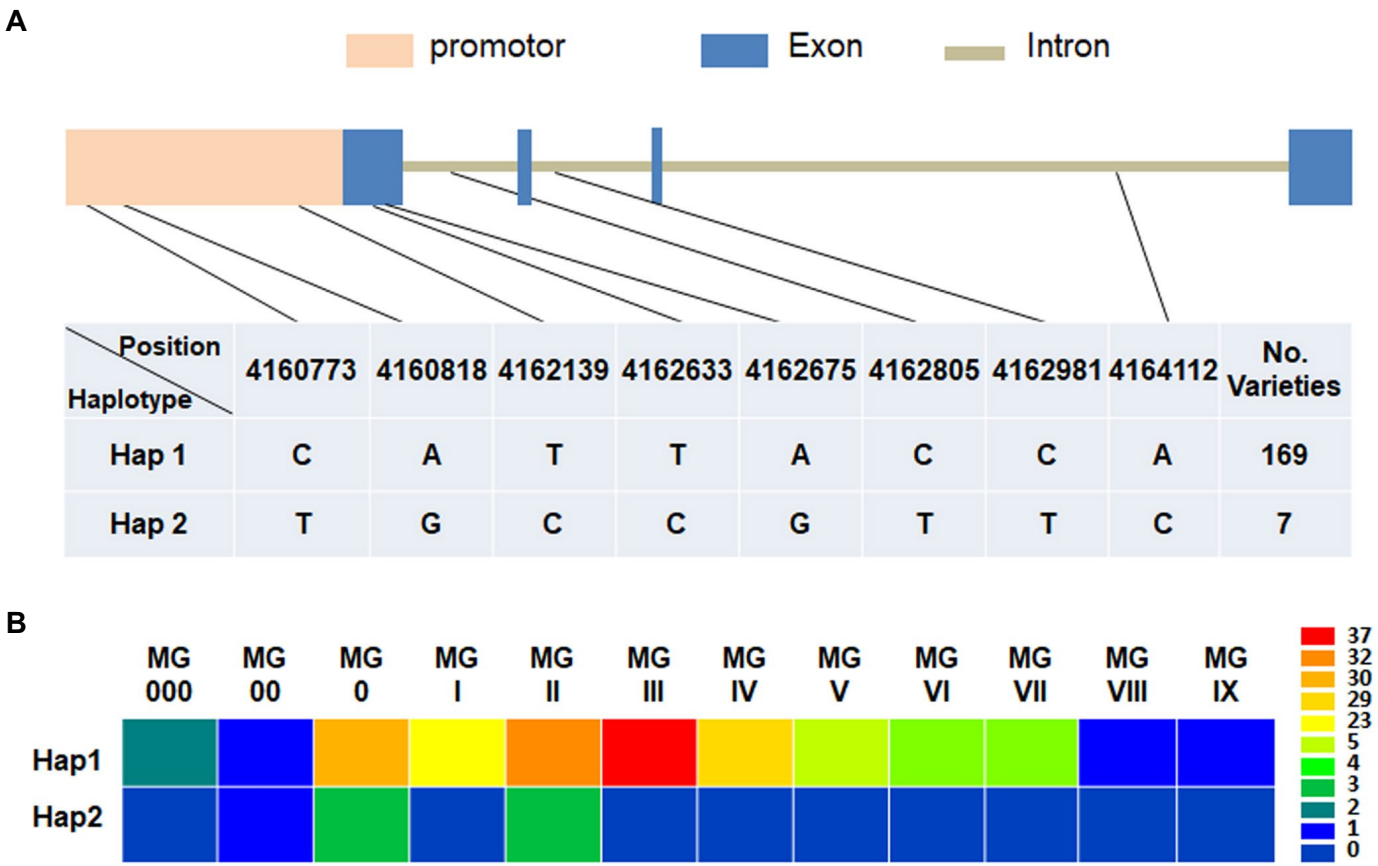
Haplotype identification **(A)** and corresponding maturity groups **(B)** of *GmFT3a* in 176 representative soybean varieties.

Based on the re-sequencing data, the natural variations of *GmFT3a* in 176 soybean varieties with diverse maturity groups and origins were analyzed and a total of 8 SNPs including 3 alleles in the promoter region (Gm16:4160524.0.4162523) and 5 alleles in genomic region (Gm16:4162524.0.4164824) were identified (Figure 7). Among the two major haplotypes, Hap1 was detected in 169 varieties ranging from MG 000 to MG IX, and Hap2 was detected in 7 varieties with MG 00 to MG II (Figure 7; Supplementary Table S2), indicating that Hap2 would be related to early maturity. Almost all Hap2 varieties collected from the high-latitude regions in northeast China, e.g., Heihe 9, Heihe 18, Heihe 38 and Huajiang 4 were bred in Heihe city (approximately 50°N), Heilongjiang Province, and the Tiefeng 18 and Tiefeng 20 varieties were bred in Tieling (approximately 42°N), Liaoning Province in northeast China.

## Discussion

### *GmFT3a* fine-tunes flowering time of soybean under LD condition

FT belongs to a phosphatidylethanolamine-binding protein family composed of six members in Arabidopsis (Kardailsky et al., 1999; Jin et al., 2021). FT is considered to be a major component of florigen and plays a critical role in the photoperiodic flowering pathway (Corbesier et al., 2007; Wigge, 2011). To date, the photoperiod-dependent flowering pathway was intensively studied and a total of 11 florigen (*FT*) homologues have been characterized in soybean (Kong et al., 2010; Sun et al., 2011; Liu et al., 2018, 2020b; Zhang et al., 2021; Su et al., 2022). *GmFT3a*, which is located on the same chromosome as the key flowering promoters *GmFT2a* and *GmFT5a*, has yet to be studied for functional identification. In this study, *GmFT3a* promoted flowering by 3.9 d in Arabidopsis under an inductive LD photoperiod, and the overexpression of *GmFT3a* in soybean resulted in earlier flowering significantly by 4.0–8.9 d than that of the control under non-inductive LD conditions (Figures 2A, 3C). Compared with other *GmFT* homologues, *GmFT2a* exhibited a stronger effect in promoting flowering than *GmFT3a*. Overexpression of *GmFT2a* resulted in earlier flowering by approximately 9 d under SD and extremely early flowering phenotypes by approximately 32 d under LD in Jack background (Cai et al., 2020); and under LD, it even drove flowering of the extreme late-maturing variety ZGDD which would have retained its vegetative growth under non-inductive LD conditions (Li et al., 2005; Sun et al., 2011). In addition, *GmFT3a* seems to have a partially similar but weaker function to *GmFT5a* and *GmFT2b*, which held dominant functions under LD condition. The overexpression of *GmFT5a* and *GmFT2b* caused earlier flowering by approximately 16 d and 7 d, respectively under LD (Chen et al., 2018; Cai et al., 2020). Besides, *GmFT3b*, which has the highest homology with *GmFT3a*, was functionally redundant in regulation of flowering time (Jiang et al., 2019; Su et al., 2022). Collectively, the supporting role of *GmFT3a* fine-tuned soybean flowering under the non-inductive LD condition.

### *GmFT3a* promoted flowering by regulating the downstream flowering-related genes

The transition from vegetative growth to reproductive growth is due to the balance of flowering activators and flowering inhibitors according to previous models for photoperiodic flowering in soybean (Liu et al., 2018; Chen et al., 2020). The overexpression of *GmFT3a* unexpectedly down-regulated that of *GmFT2a* and *GmFT5a* under LD (Figure 4), which was inconsistent with the up-regulation of *GmFT2a* and *GmFT5a* by overexpression of *GmFT2b* (Chen et al., 2020). Under LD conditions, higher expression levels of flowering activators are required to overcome the enhanced the effects of flowering inhibitors. This implied that the function of *GmFT3a* might differ from that of *GmFT2b* in flowering regulation, and the lowered expression of *GmFT2a* and *GmFT5a* is probably responsible for the minor/weak effect on promoting the flowering of *GmFT3a*. *GmFT3b* was up-regulated under the overexpression of *GmFT3a* (Figure 4), and a previous study showed that *GmFT3a* and *GmFT3b* were both significantly up-regulated in the *ft2a* and *ft5a* single soybean mutant, respectively (Li et al., 2021), suggesting that this pair of homologues might share a similar trend under the lack of major promoting effector *GmFT2a* and *GmFT5a*. However, there was no significant change in the expression of *GmFT3a* displayed in both overexpression of *GmFT3b* and the *ft3b* mutant (Su et al., 2022). It could be inferred that *GmFT3a* probably functioned upstream on the *GmFT3b*, and the hypothesis still needs more molecular evidence and flowering phenotype of the *GmFT3a* mutant. In addition, higher expression of *GmFT3a* was detected in the relatively early maturing varieties (MG 000 and MG 00) under SD, suggesting that *GmFT3a* might correlate with early flowering and function as an effective promoter of flowering in non-inductive photoperiodic conditions (Jia et al., 2014; Cao et al., 2016b; Liu et al., 2020b).

In Arabidopsis, the expression of the *AtFT*, *AtSOC1* and *AtCO* genes was significantly increased in the overexpression of *GmFT3a* (*p* < 0.05, Figure 2D). A similar pattern was detected in the overexpression of *GmFT2a* Arabidopsis, which directed the up-regulation of *AtFT* and *AtSOC1* (Sun et al., 2011). The increase in *AtSOC1* in the age pathway was potentially involved in flowering signal integration (Srikanth and Schmid, 2011; Lv et al., 2021). The RNA transcripts of floral identity genes, including *GmAP1*, *GmSOC1*, *GmLFY* and *GmFDL19*, were increased in *GmFT2a-* or *GmFT5a*-overexpressing soybean lines compared with the wild type (Nan et al., 2014; Takeshima et al., 2019).

Among the transcriptomic DEGs, the large number under LD conditions was more than 3-fold compared to SD conditions (Table 1; Figure 5). The functional patterns of *GmFT3a* were divided into two main aspects: the vegetative growth accelerator and signal transduction pathway. *GmFT3a* probably achieved its promotional roles through the enhancement of genes involved in cell division, photosynthesis, carbohydrate metabolism, fatty acid biosynthesis, and ascorbate metabolism. Moreover, the genes encoding signaling regulation and transcription factors, such as ethylene-responsive transcription factor *AP2-EREBP*, auxin response factor *ARF*, zinc finger protein *COL16*, MADS-box family, and *GRF4* (*GROWTH-REGULATING FACTOR 4*), were involved in plant growth and metabolisms (Supplementary Table S3). These differentially expressed transcription factors were significantly correlated with plant signaling and the regulation of flowering development (Ratcliffe and Riechmann, 2002; Matias-Hernandez et al., 2016; Xu et al., 2021a). Furthermore, the KEGG pathway maps provided abundant information on the exploration of signaling and metabolic pathways involved in *GmFT3a*. Many differentially expressed unigenes were enriched in signaling (Plant hormone signal transduction pathway) under LD conditions and metabolic processes (Biosynthesis of secondary metabolites) under SD conditions, respectively. Transcriptome analysis revealed *GmFT3a* might promote flowering by regulating reproductive signals and growth regulators. However, the functional complementarity and interactions among multiple *FT* homologues remain to be further verified (Lee et al., 2021). Actually, the interaction and allocation mechanism among the *GmFT* family genes will be further certificated confirmed by evaluation of the phenotypes of the *Gmft3a* mutant and/or the double mutant of different combinations of *Gmft* members in future studies.

### The natural variations of *GmFT3a* are related to the high-latitude adaptability of soybean

The adaptation of soybean varieties to different latitudes or daylengths was mainly attributed to the combinations and functional diversifications among the photoperiod-related genes (Liu et al., 2020b; Lin et al., 2021b). The wide distribution of soybean varieties results from rich natural variations and different combinations of genes and QTLs controlling flowering time (Watanabe et al., 2012; Liu et al., 2022). The early maturity of soybeans in Northeast China was largely attributed to local exotic migration and selection against positive alleles causing new recombination, while only few new allele emergence/mutation occurred (Fu et al., 2020). Therefore, allelic variations in flowering time-related genes could be applied as an effective means for breeding (Cao et al., 2016b; Khan et al., 2022). There were natural variations within the *GmFT* family genes that contribute to soybean adaptation to various growing areas (Jiang et al., 2019). Some natural variations in *GmFT2a* caused significantly late flowering, which is suitable for soybean accessions which adapted to low-latitude areas (Li et al., 2021). In contrast, some single-nucleotide polymorphisms (SNPs) in *GmFT5a* result in early flowering, which was preferred in high-latitude areas (Takeshima et al., 2016). For *GmFT3a*, multiple SNP alleles in both promoter and genomic regions suggested that genomic variations and regulation of gene expression of *GmFT3a* both contributed to the flowering and maturity diversities (Figure 7). Notably, the rare Hap2 of *GmFT3a* showed relatively early maturity and most of these varieties were bred for high-latitude regions in China, suggesting that *GmFT3a*-Hap2 may have contributed to the adaptation of soybean to higher latitude areas. For soybean breeding, it is an effective way to broaden the adaptive region of soybean varieties through recombining and modifying genes in photoperiod pathway, e.g., various combinations of mutations at the *E* loci (*E1*, *E2*, *E3* and *E4*) provide considerable genetic plasticity that contributes to soybean cultivation at diverse latitudes (Jiang et al., 2014; Liu et al., 2020c, 2022); *J* (the major gene for adaptation to short-day and high temperature conditions at low latitudes; Yue et al., 2016; Lu et al., 2017), *GmPRR37* (Wang et al., 2020), the *ft2aft5a* double mutants showed late flowering and produced more pods and seeds under SD conditions (Cai et al., 2020). When using these genes with large impacts on flowering time and maturity, it might be difficult to maintain the original yield and quality traits of elite varieties. However, fine-tuning florigen expression is a promising strategy for high yield (Xu et al., 2021b). In this study, we found that the overexpression of *GmFT3a* promoted flowering by approximately 4.0–8.9 d under LD conditions, suggesting that *GmFT3a* provides an opportunity to lightly modify the flowering time and maturity of soybean varieties, which is helpful to retain yield and agronomic traits of an elite variety with extension of its adaptive regions (Qi et al., 2021).

## Data availability statement

The datasets presented in this study can be found in online repositories. The names of the repository/repositories and accession number(s) can be found at: https://www.ncbi.nlm.nih.gov/, PRJNA832118.

## Author contributions

TH and LY conceived the study. YW and CZ performed the Arabidopsis transformation experiment. LC and WH performed the soybean transformation. JW and LZ recorded the phenotype of transgenic soybean. SS and CW provided and maintained the MG standard varieties. TW and BJ analyzed the re-sequencing data. YC and FC performed the gene expression experiment. SY, XX, and WS analyzed the transcriptomic data. SY and YW drafted the manuscripts. All authors contributed to the article and approved the submitted version.

## Funding

This work was supported by grants from National Natural Science Foundation of China (32001566), the Central Public-interest Scientific Institution Basal Research Fund (No. Y2022GH08), and the China Agriculture Research System (CARS-04).

## Conflict of interest

The authors declare that the research was conducted in the absence of any commercial or financial relationships that could be construed as a potential conflict of interest.

## Publisher’s note

All claims expressed in this article are solely those of the authors and do not necessarily represent those of their affiliated organizations, or those of the publisher, the editors and the reviewers. Any product that may be evaluated in this article, or claim that may be made by its manufacturer, is not guaranteed or endorsed by the publisher.
