# Supplementary material for: GmFT3a fine-tunes flowering time and improves adaptation of soybean to higher latitudes
Source: Front Plant Sci. 2022 Jul 25;13:929747. doi: 10.3389/fpls.2022.929747 (PMC9358591; doi:10.3389/fpls.2022.929747)
Supplement: Supplementary file 1 [file Data_Sheet_1.docx]

## Supplementary Information


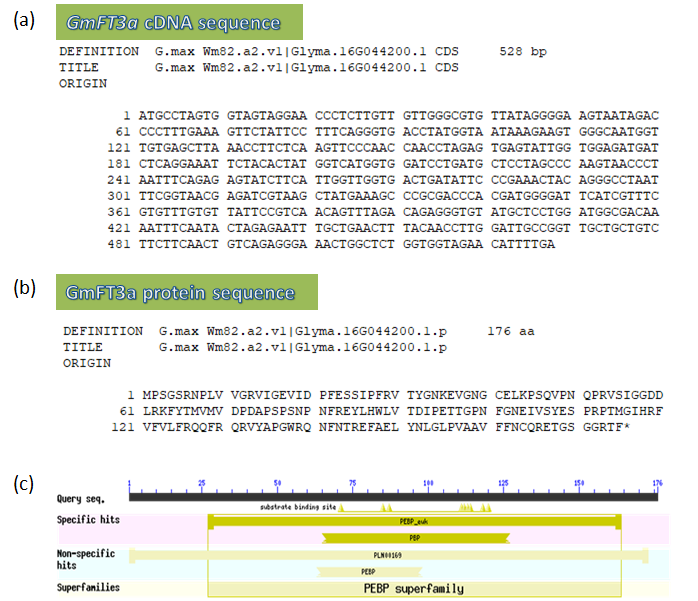


Fig. S1 The *GmFT3a* cDNA (a), GmFT3a protein sequences (b) and the structure information (c).


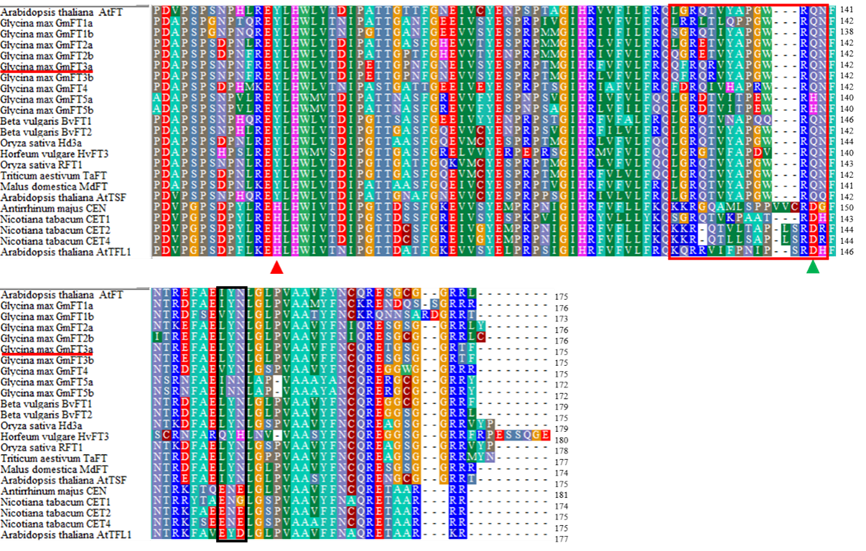


Fig. S2 Sequences alignment of FT/TFL1 family members from flowering plants.

The red and green triangle indicate the Tyr85/His88 and Asp144/Gln140 residues distinguishing FT from TFL1, respectively. 14-amino-acid external loop is boxed in red; the VYN triad is boxed in black.


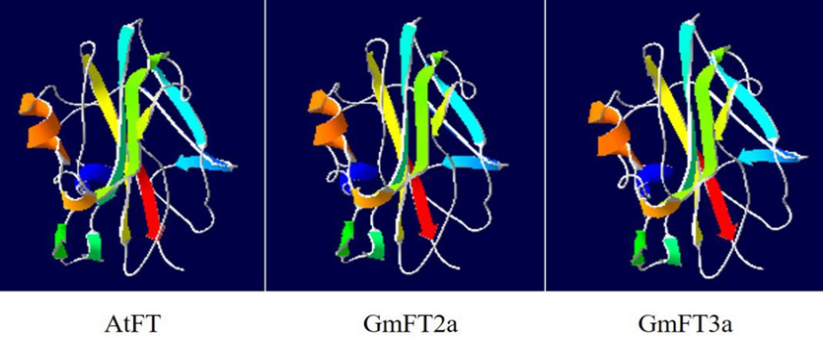


Fig. S3 The protein structure prediction of AtFT, GmFT2a and GmFT3a.


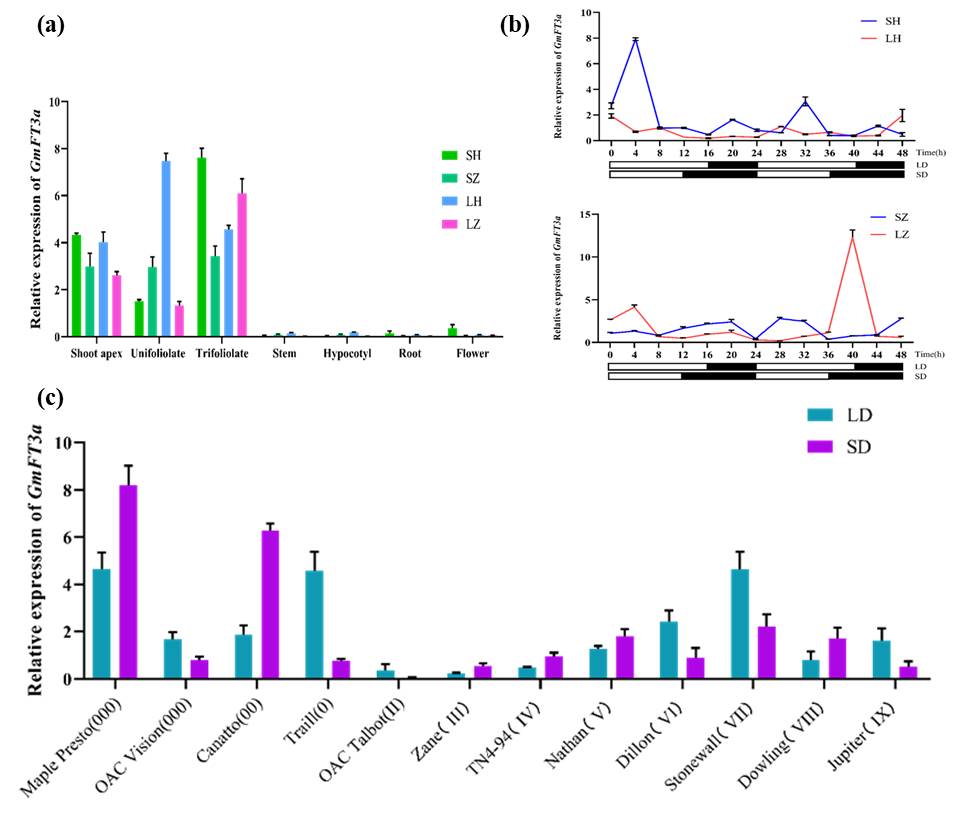


Fig. S4. *GmFT3a* expression patterns in different organs (a), photoperiodic treatments (b) and 12 varieties from different maturity groups of soybeans on 13 d under the different photoperiodic treatments (c). Note: SH, HH27 in SD; SZ, ZGDD in SD; LH, HH27 in LD; LZ, ZGDD in LD. Blue lines indicate in SD; Red lines indicate in LD; The numbers in the bracket denote the MG group for each variety. Samples (unifoliate leaves) were collected 4 h after the light was tuned on. SD: 12/12h light/dark; LD: 16/8h light/dark.

Table S1 The primers used in this study

| Primer Name | Primer Sequence | Note |
| --- | --- | --- |
| *GmFT3a*-F | ATGCCTAGTGGTAGTAGGAACCC | Cloning of GmFT3a-forward |
| *GmFT3a*-R | TCAAAATGTTCTACCACCAGAGCC | Cloning of GmFT3a-reverse |
| Sub-*GmFT3a*-F | GTCGACATGCCTAGTGGTAGTAGGA | Subcloning of *GmFT3a*-forward |
| Sub-*GmFT3a*-R | TCTAGAAAATGTTCTACCACCAGAG | Subcloning of *GmFT3a*-reverse |
| *qGmActin*-F | CGGTGGTTCTATCTTGGCATC | Internal reference *GmActin* qRT-PCR primer-forward |
| *qGmActin*-R | GTCTTTCGCTTCAATAACCCTA | Internal reference *GmActin* qRT-PCR primer-reverse |
| *qGmFT1a*-F | ATTCCTGCAACTACAGGGGC | *GmFT1a* qRT-PCR primer-forward |
| *qGmFT1a*-R | AAGTACATGGCCGCTACTGG | *GmFT1a* qRT-PCR primer-reverse |
| *qGmFT1b*-F | TGCACCTAGCCCAGGAAATC | *GmFT1a* qRT-PCR primer-forward |
| *qGmFT1b*-R | CCATCATCGGTCGTGGACTC | *GmFT1a* qRT-PCR primer-reverse |
| *qGmFT2a*-F | ATGCACCTAGCCCAAGTGAC | *GmFT2a* qRT-PCR primer-forward |
| *qGmFT2a*-R | TACACGGTCTCCCTACCCAG | *GmFT2a* qRT-PCR primer-reverse |
| *qGmFT3a*-F | AGCGCCCAAATAAGAAAGGT | *GmFT3a* qRT-PCR primer-forward |
| *qGmFT3a*-R | TGGTACATATGAGTGCTTCGGT | *GmFT3a* qRT-PCR primer-reverse |
| *qGmFT3b*-F | TAATTTCGGTAACGAGGTTGT | *GmFT3b* qRT-PCR primer-forward |
| *qGmFT3b*-R | AAAATGTTCTACCACCAGAGCC | *GmFT3b* qRT-PCR primer-reverse |
| *qGmFT5a*-F | CACGGGAGAACCCTCTTGTTAT | *GmFT5a* qRT-PCR primer-forward |
| *qGmFT5a*-R | GGTCTTCACCACCAACAGTAACC | *GmFT5a* qRT-PCR primer-reverse |
| *qAtActin*-F | AAGTCTTGTTCCAGCCCTCG | Internal reference *AtActin* qRT-PCR primer-forward |
| *qAtActin*-R | TTTGCTCATACGGTCAGCGA | Internal reference *AtActin* qRT-PCR primer-reverse |
| *qAtFT*-F | CCAAGTCCCACTGCAGGAAT | *AtFT* qRT-PCR primer-forward |
| *qAtFT*-R | AAGTCTTCTTCCTCCGCAGC | *AtFT* qRT-PCR primer-reverse |
| *qAtCO*-F | GAACGCCCAAAGGGACAGTA | *AtCO* qRT-PCR primer-forward |
| *qAtCO*-R | CCGCGGTCTTATCTCTGCAT | *AtCO* qRT-PCR primer-reverse |
| *qAtSOC1*-F | GGTGAGGGGCAAAACTCAGA | *AtSOC1* qRT-PCR primer-forward |
| *qAtSOC1*-R | TGCATATTGGAGCTGGCGAA | *AtSOC1* qRT-PCR primer-reverse |
| *qAtSVP*-F | AAACTGGTTTGACGCGTGTG | *AtSVP* qRT-PCR primer-forward |
| *qAtSVP*-R | TTCTCCGATTCAGCACCACC | *AtSVP* qRT-PCR primer-reverse |

Table S2 The haplotype classification of *GmFT3a* and maturity groups of representive soybean cultivars

| **Variety** | **Country** | **Maturity Group** | **Haplotype of *GmFT3a*** |
| --- | --- | --- | --- |
| Dongnong 4 | China | MG 000 | Hap 1 |
| Beihudou | China | MG 000 | Hap 1 |
| Heihe 3 | China | MG 00 | Hap 1 |
| 75-158 | America | MG 0 | Hap 1 |
| Yudou 8 | China | MG 0 | Hap 1 |
| Heihe 27 | China | MG 0 | Hap 1 |
| Jingshanpu | China | MG 0 | Hap 1 |
| Hejiao 6 | China | MG 0 | Hap 1 |
| Suinong 3 | China | MG 0 | Hap 1 |
| Mancangjin | China | MG 0 | Hap 1 |
| Gefeng 22 | China | MG 0 | Hap 1 |
| Beifeng 11 | China | MG 0 | Hap 1 |
| Fengshou 24 | China | MG 0 | Hap 1 |
| Zhi 2 | China | MG 0 | Hap 1 |
| Clay | America | MG 0 | Hap 1 |
| Heihe 54 | China | MG 0 | Hap 1 |
| Wilkin | America | MG 0 | Hap 1 |
| Heihe 19 | China | MG 0 | Hap 1 |
| Heihe 51 | China | MG 0 | Hap 1 |
| Mengdou 30 | China | MG 0 | Hap 1 |
| Fengshou 19 | China | MG 0 | Hap 1 |
| Beidou 5 | China | MG 0 | Hap 1 |
| Dengke 1 | China | MG 0 | Hap 1 |
| Gefeng 25 | China | MG 0 | Hap 1 |
| Fengshou 17 | China | MG 0 | Hap 1 |
| Heilongjiang 41 | China | MG 0 | Hap 1 |
| Merit | America | MG 0 | Hap 1 |
| Fengshou 10 | China | MG 0 | Hap 1 |
| Beifeng 9 | China | MG 0 | Hap 1 |
| Kangxian 4 | China | MG 0 | Hap 1 |
| Dongnong 72-806 | China | MG 0 | Hap 1 |
| Fengshou 12 | China | MG 0 | Hap 1 |
| Gefeng 35 | China | MG 0 | Hap 1 |
| Suinong 14 | China | MG I | Hap 1 |
| Evans | America | MG I | Hap 1 |
| Gefeng 47 | China | MG I | Hap 1 |
| Gefeng 50 | China | MG I | Hap 1 |
| Heinong 35 | China | MG I | Hap 1 |
| Suinong 8 | China | MG I | Hap 1 |
| Gefeng 55 | China | MG I | Hap 1 |
| Suinong 28 | China | MG I | Hap 1 |
| Suinong 10 | China | MG I | Hap 1 |
| Heinong 37 | China | MG I | Hap 1 |
| Changjihuangdou | China | MG I | Hap 1 |
| Heinong 44 | China | MG I | Hap 1 |
| Taixingheidou | China | MG I | Hap 1 |
| Edou 8 | China | MG I | Hap 1 |
| Heinong 16 | China | MG I | Hap 1 |
| Heinong 48 | China | MG I | Hap 1 |
| Heinong 33 | China | MG I | Hap 1 |
| Heinong 43 | China | MG I | Hap 1 |
| Hark | America | MG I | Hap 1 |
| Heinong 26 | China | MG I | Hap 1 |
| Jidou 7 | China | MG I | Hap 1 |
| Changnong 4 | China | MG I | Hap 1 |
| Jilin 4 | China | MG I | Hap 1 |
| Jilin 20 | China | MG II | Hap 1 |
| Zihua 4 | China | MG II | Hap 1 |
| Jiti 5 | China | MG II | Hap 1 |
| Fushou | China | MG II | Hap 1 |
| Jiunong 9 | China | MG II | Hap 1 |
| Tiefeng 19 | China | MG II | Hap 1 |
| Jilin 13 | China | MG II | Hap 1 |
| Huangbaozhu | China | MG II | Hap 1 |
| Xiaojinhuang 1 | China | MG II | Hap 1 |
| Zaofeng 1 | China | MG II | Hap 1 |
| Jiyu 57 | China | MG II | Hap 1 |
| Jilin 30 | China | MG II | Hap 1 |
| Kaiyo 8 | China | MG II | Hap 1 |
| Tiefeng 3 | China | MG II | Hap 1 |
| Changnong 5 | China | MG II | Hap 1 |
| Kaiyu 3 | China | MG II | Hap 1 |
| Beeson | America | MG II | Hap 1 |
| Fengchengniupidou | China | MG II | Hap 1 |
| Jiunong 22 | China | MG II | Hap 1 |
| Jiti 1 | China | MG II | Hap 1 |
| Amsoy 71 | America | MG II | Hap 1 |
| Tianlong 1 | China | MG II | Hap 1 |
| Fengdihuang | China | MG II | Hap 1 |
| Tiefeng 8 | China | MG II | Hap 1 |
| Jilin 3 | China | MG II | Hap 1 |
| Aijiaozao | China | MG II | Hap 1 |
| Jihuang 10 | China | MG II | Hap 1 |
| Jindou 2 | China | MG II | Hap 1 |
| Kaiyo 10 | China | MG II | Hap 1 |
| Jin 6604-24 | China | MG II | Hap 1 |
| Amsoy | America | MG II | Hap 1 |
| Harosoy 63 | America | MG II | Hap 1 |
| Shangcaiercaopingdingshi | China | MG III | Hap 1 |
| Shangyukanshanbai | China | MG III | Hap 1 |
| Xiangdou 3 | China | MG III | Hap 1 |
| Wenfeng 5 | China | MG III | Hap 1 |
| Dandou 4 | China | MG III | Hap 1 |
| Zhechun 3 | China | MG III | Hap 1 |
| Dandou 2 | China | MG III | Hap 1 |
| Zhonghuang 35 | China | MG III | Hap 1 |
| Zhonghuang 30 | China | MG III | Hap 1 |
| Wenfeng 7 | China | MG III | Hap 1 |
| Zhengzhou 135 | China | MG III | Hap 1 |
| Huairouhuangdou | China | MG III | Hap 1 |
| Jin 33 | China | MG III | Hap 1 |
| Jindou 25 | China | MG III | Hap 1 |
| Handou 5 | China | MG III | Hap 1 |
| Liaodou 15 | China | MG III | Hap 1 |
| Ludou 4 | China | MG III | Hap 1 |
| Wayne | America | MG III | Hap 1 |
| Kefeng 6 | China | MG III | Hap 1 |
| Jin8-14 | China | MG III | Hap 1 |
| Jindou 19 | China | MG III | Hap 1 |
| Tiefeng 31 | China | MG III | Hap 1 |
| Zhonghuang 37 | China | MG III | Hap 1 |
| Xudou 9 | China | MG III | Hap 1 |
| Zhongdou 19 | China | MG III | Hap 1 |
| Weiqingdou | China | MG III | Hap 1 |
| Heibiqing | China | MG III | Hap 1 |
| Hezeniumaohuang | China | MG III | Hap 1 |
| Zhonghuang 13 | China | MG III | Hap 1 |
| Yidoupingdinghuang | China | MG III | Hap 1 |
| Qunyingdou | China | MG III | Hap 1 |
| Jidou 12 | China | MG III | Hap 1 |
| Guichun 1 | China | MG III | Hap 1 |
| Yanhuang 1 | China | MG III | Hap 1 |
| Hedou 13 | China | MG III | Hap 1 |
| Fengshouhuang | China | MG III | Hap 1 |
| Jidou 17 | China | MG III | Hap 1 |
| Jinningdahuangdou | China | MG IV | Hap 1 |
| Jindou 21 | China | MG IV | Hap 1 |
| Tiefeng 29 | China | MG IV | Hap 1 |
| Haiyangpamanqing | China | MG IV | Hap 1 |
| Yuejin 5 | China | MG IV | Hap 1 |
| Clark | America | MG IV | Hap 1 |
| Youbian 30 | China | MG IV | Hap 1 |
| Qiandou 6 | China | MG IV | Hap 1 |
| Xudou 2 | China | MG IV | Hap 1 |
| Yudou 2 | China | MG IV | Hap 1 |
| Zheng 92116 | China | MG IV | Hap 1 |
| Xudou 1 | China | MG IV | Hap 1 |
| Cutler | America | MG IV | Hap 1 |
| Yudou 22 | China | MG IV | Hap 1 |
| Chenliuniumaohuang | China | MG IV | Hap 1 |
| Changpingqingdou | China | MG IV | Hap 1 |
| Xudou 5 | China | MG IV | Hap 1 |
| Yuejin 4 | China | MG IV | Hap 1 |
| Baihuadou | China | MG IV | Hap 1 |
| Dandou 1 | China | MG IV | Hap 1 |
| Guichun 8 | China | MG IV | Hap 1 |
| Naiyinheidou | China | MG IV | Hap 1 |
| Jindou 23 | China | MG IV | Hap 1 |
| Bahong 1 | China | MG IV | Hap 1 |
| Yunyizao | China | MG IV | Hap 1 |
| Liuyuehuang | China | MG IV | Hap 1 |
| Jinjiangdaqingren | China | MG IV | Hap 1 |
| Zigongqingpidou | China | MG IV | Hap 1 |
| 77-14 | America | MG IV | Hap 1 |
| Houzimao | China | MG V | Hap 1 |
| Edou 2 | China | MG V | Hap 1 |
| Baishuidou | China | MG V | Hap 1 |
| Yulindahuangdou | China | MG V | Hap 1 |
| Bedford | America | MG V | Hap 1 |
| Juhuangdadou | China | MG VI | Hap 1 |
| Suxie1 | China | MG VI | Hap 1 |
| Tracy | America | MG VI | Hap 1 |
| Centennial | America | MG VI | Hap 1 |
| Braxton | America | MG VII | Hap 1 |
| Pingguohuangdou | China | MG VII | Hap 1 |
| Shangraodaqingsi | China | MG VII | Hap 1 |
| Qiudou 1 | China | MG VII | Hap 1 |
| Lanxidaqingdou | China | MG VIII | Hap 1 |
| Zigongdongdou | China | MG IX | Hap 1 |
| Heihe 9 | China | MG 00 | Hap 2 |
| Heihe 18 | China | MG 0 | Hap 2 |
| Huajiang 4 | China | MG 0 | Hap 2 |
| Heihe 38 | China | MG 0 | Hap 2 |
| Tiefeng 20 | China | MG II | Hap 2 |
| Tiefeng 18 | China | MG II | Hap 2 |
| Ludou 11 | China | MG II | Hap 2 |
| Maple Prestro | America | MG 0 | - |
| OAC Version | America | MG 0 | - |
| Canatto | America | MG 0 | - |
| Traill | America | MG 0 | - |
| OAC Talbot | America | MG II | - |
| Zane | America | MG III | - |
| TN4-94 | America | MG IV | - |
| Nathan | America | MG V | - |
| Dillon | America | MG VI | - |
| Stonewall | America | MG VII | - |
| Dowling | America | MG VIII | - |
| Jupiter | America | MG X | - |

Table S3 Diferential expression of transcription factor genes between *GmFT3a* transgenic

soybean and the wildtype

| **Family** | **Gene ID** | **Gene annotation** | **Difference in expression*** | **P value (adjusted )** |
| --- | --- | --- | --- | --- |
| ABI3VP1 | Glyma.11G124100 | B3 domain-containing VRN1 | 1.125 | 0.036 |
| AP2-EREBP | Glyma.20G155200 | Ethylene-responsive ERF025 | 6.433 | 0.023 |
|  | Glyma.06G221800 | Ethylene-responsive WIN1 | 5.988 | 0.001 |
|  | Glyma.17G131900 | Dehydration-responsive element-binding protein 1C | 5.793 | 0.001 |
| ARF | Glyma.07G202200 | ARFC | 2.505 | <0.001 |
|  | Glyma.14G217700 | ARFE | 2.298 | 0.033 |
|  | Glyma.13G174000 | ARFC | 2.244 | <0.001 |
| bHLH | Glyma.08G271000 | bHLH96 | 5.444 | <0.001 |
|  | Glyma.15G104600 | MUTE | 4.996 | 0.012 |
|  | Glyma.07G049100 | bHLH67 | 3.462 | <0.001 |
| bZIP | Glyma.13G050700 | BZP43 | 4.488 | 0.029 |
|  | Glyma.12G208400 | BZP34 | 2.946 | 0.007 |
|  | Glyma.14G071400 | bZIP 11 | 2.846 | <0.001 |
| C2C2-CO-like | Glyma.07G091400 | COL16 | -1.411 | <0.001 |
|  | Glyma.09G184600 | COL16 | -1.863 | <0.001 |
| C2C2-Dof | Glyma.06G182200 | DOF5.6 | 3.963 | <0.001 |
|  | Glyma.05G037800 | DOF5.6 | 2.899 | <0.001 |
| C2H2 | Glyma.03G173300 | Zinc finger protein ZAT11 | 4.948 | 0.031 |
|  | Glyma.03G157400 | Zinc finger protein MAGPIE | 4.090 | <0.001 |
| G2-like | Glyma.04G035700 | KAN4 | 3.283 | 0.001 |
| GRAS | Glyma.11G016100 | Scarecrow-like protein 3 | -2.130 | 0.005 |
| GRF | Glyma.10G067200 | Growth-regulating factor 4 | 4.644 | 0.014 |
| MADS | Glyma.13G256900 | MADS-box protein SVP | 6.137 | <0.001 |
|  | Glyma.07G181600 | MADS-box protein SVP | 5.284 | <0.001 |
|  | Glyma.08G065300 | MADS-box 27 | 4.997 | <0.001 |
|  | Glyma.15G058000 | MADS-box JOINTLESS | 3.425 | <0.001 |
| MYB | Glyma.02G013900 | MYB12 | 7.394 | <0.001 |
|  | Glyma.09G038900 | MYB111 | 6.509 | <0.001 |
| NAC | Glyma.05G202300 | NAC 83 | 4.487 | <0.001 |
|  | Glyma.18G043900 | NAC86 | 4.235 | 0.017 |
|  | Glyma.05G195000 | NAC2 | 4.144 | 0.030 |
| TCP | Glyma.18G121400 | TCP15 | 6.635 | <0.001 |
|  | Glyma.06G284500 | TCP4 | 2.349 | <0.001 |
| WRKY | Glyma.17G222300 | WRKY transcription factor 40 | 6.470 | 0.009 |
| zf-HD | Glyma.02G211900 | ZHD2_Zinc-finger homeodomain protein 2 | 5.035 | <0.001 |
